# Case Report and retrospective literature analysis of pediatric lower esophageal squamous cell carcinoma: focus on diagnostic challenges and therapeutic strategies

**DOI:** 10.3389/fped.2026.1777348

**Published:** 2026-04-30

**Authors:** Jiani Hu, Xianlan Xiang, Yichao Zhou, Peng Cai, Menglei Zhu, Jie Zhu

**Affiliations:** 1 Department of General Surgery, Children’s Hospital of Soochow University, Suzhou, Jiangsu, China; 2 Department of Pediatric Surgery, Wanzhou District Maternal and Child Health Hospital of Chongqing, Chongqing, China

**Keywords:** case report, children, diagnostic and therapeutic strategies, esophageal squamous cell carcinoma, literature review

## Abstract

Malignant solid tumors of the gastrointestinal tract are clinically rare in children, with malignant solid tumors of the esophagus being particularly uncommon. Clinical reports on esophageal cancer in children are extremely scarce. This report describes a case of lower esophageal squamous cell carcinoma in a 10-year-old male child. By integrating the clinical management process and relevant literature, we analyze the clinical characteristics, diagnostic and therapeutic key points, and prognostic factors of pediatric esophageal squamous cell carcinoma, aiming to provide reference for its clinical management. The patient was admitted for “vomiting for 1 month” and diagnosed with stage IIIA (T1N2M0) squamous cell carcinoma of the lower esophagus. Despite treatment including chemotherapy and laparoscopic gastrostomy, the disease progressed, and the patient died 7 months after diagnosis. Pediatric esophageal squamous cell carcinoma is often diagnosed at an advanced stage with poor prognosis. Its clinical manifestations lack specificity, and no definitive treatment protocol currently exists. It differs significantly from adult tumors in clinical presentation and management strategies, not merely representing a “miniature version” of adult tumors. Currently, no standardized treatment exists for pediatric esophageal squamous cell carcinoma. Available evidence, derived primarily from adult studies and isolated pediatric case reports, suggests that a comprehensive approach combining neoadjuvant chemoradiotherapy with surgery may be cautiously considered in selected pediatric patients. Delaying surgery until tumor downstaging and optimal surgical timing are achieved may potentially improve survival outcomes for these patients.

## 1 Introduction

Malignant solid tumors of the gastrointestinal tract are remarkably uncommon in pediatric populations, with esophageal malignant solid tumors representing an even rarer subset (1, 2). pediatric esophageal cancer, in particular, has been sparsely documented in clinical literature, presenting substantial challenges to clinicians in terms of early detection, precise diagnosis, and effective therapeutic management. In contrast, esophageal cancer is a globally prevalent gastrointestinal malignancy in adults, ranking eleventh in incidence and seventh in mortality across all cancers worldwide. It is important to emphasize that these epidemiological data primarily reflect adult disease; the epidemiology of pediatric esophageal cancer is fundamentally different, with extremely low incidence and distinct biological behavior.

Due to its low prevalence, pediatric cases lack standardised diagnostic criteria and treatment protocols, frequently resulting in initial misdiagnosis. Its non-specific clinical manifestations—including vomiting, retrosternal discomfort, and weight loss—overlap with common pediatric gastrointestinal conditions such as gastritis or reflux oesophagitis (1). Crucially, pediatric tumors are not mere “miniature versions” of adult counterparts, whereas direct evidence specific to pediatric esophageal cancer remains extremely limited. They diverge substantially in clinical presentation, biological behaviour, and treatment responsiveness, necessitating customised approaches that accommodate children's unique physiological characteristics (3, 4).

Herein, we report a detailed case of a 10-year-old male diagnosed with stage IIIA (T1N2M0) lower esophageal squamous cell carcinoma, supplemented by a systematic literature review. Our objective is to characterise the key clinical attributes, diagnostic challenges, and therapeutic complexities of this condition, while evaluating the feasibility of adapting adult multidisciplinary treatment frameworks to this vulnerable pediatric cohort. We aim to provide actionable insights that could refine clinical practice and enhance outcomes for this rare pediatric malignancy.

## 2 Case report

The patient, a 10-year-old boy, was admitted to the Department of Gastroenterology at our hospital on February 22, 2020, presenting with “vomiting for one month.” Since symptom onset, the patient experienced vomiting several to over ten times daily. Episodes were predominantly non-projectile vomiting after meals, with vomitus consisting of ingested food without bile. Occasional blood streaks were noted during frequent vomiting episodes. Symptoms included retrosternal discomfort, back soreness (more pronounced in supine position, alleviated in sitting), Occasional mild abdominal pain, primarily in the upper abdomen, was reported. No diarrhea or fever was present. The patient had multiple outpatient visits at a local hospital during the illness. Ultrasound examinations revealed no significant abnormalities in the digestive tract. Treatment with acid-suppressing and gastric-protective agents (omeprazole, aluminum carbonate magnesium) and prokinetic agents failed to control the frequent vomiting. The patient's mental status and sleep are acceptable, but appetite is poor. Stools occur every 2–3 days and are dark brown. Urination shows no significant abnormalities. Weight loss of approximately 5 kg has occurred over the past month. The patient previously had a generally good constitution. Both parents are in good health, and there is no family history of similar hereditary diseases.

### 2.1 Physical examination

T 36.2 °C, *P* 108 bpm, R 26 bpm, Wt 30.5 kg, H 145 cm, SpO2 97% (without supplemental oxygen), BP 120/90 mmHg, no significant findings. Preliminary Diagnosis: Vomiting of unknown origin (Gastritis? Reflux esophagitis? Esophageal stricture? Achalasia?).

### 2.2 Laboratory tests

February 22, 2020 Complete Blood Count and CRP: WBC: 8.04 × 10^9^/L, High-Sensitivity CRP: 11.34 mg/L, Lymphocytes: 20.4%, Hemoglobin: 134 g/L, Platelet Count: 385 × 10^9^/L, Neutrophils: 72.2%; Urinalysis and sediment: Epithelial cells: 12.2/*μ*L, Ketones: 100 mg/dL (+++), Bacteria: 57.5/μL, Mucus: 53.17/μL; Urine amylase: 648.1 U/L; Complete Biochemistry and Humoral Immunity: Complement C4: 0.54 g/L, Creatinine: 38 μmol/L, Immunoglobulin A: 2.09 g/L, Immunoglobulin G: 12.57 g/L, Urea nitrogen: 278 mmol/L; Uric acid: 481 μmol/L; Prealbumin: 122 mg/L; Total cholesterol: 5.61 mmol/L; Tumor marker panel: Alpha-fetoprotein: 21.67 μg/L, Carbohydrate Antigen CA125: 61.5 μg/mL, Neuron Enolase: 21.07 ng/mL. Routine stool examination and occult blood test, serum amylase, pre-transfusion panel, hepatitis B virus DNA, coagulation profile, hepatitis B serology (three markers), lymphocyte subpopulations, cardiac panel (three markers), blood gas analysis, and electrolytes showed no significant abnormalities.

### 2.3 Imaging findings

February 22, 2020 Routine gastroscopy revealed a lower esophageal ulcer (nature to be determined); Chest x-ray showed increased pulmonary and haziness; Abdominal and pelvic CT revealed thickening of the distal esophageal wall and hypoplasia of the left kidney; Upper GI barium meal study on February 23 showed impaired barium passage in the lower esophagus and weak gastric peristaltic waves; February 24 chest CT: Thickened walls in the lower esophagus and cardia with multiple nodular shadows (possible lymph node necrosis), thickened gastric antrum wall, multiple retroperitoneal lymph nodes, hypoplastic left kidney; February 26 PET-CT: Mass lesion in the lower esophagus and gastric cardia, multiple enlarged lymph nodes in the right subclavian region, left axilla, mediastinum, and retroperitoneum (all with increased glucose metabolism). Considered possible malignant lesion in the lower esophagus and cardia with multiple lymph node metastases (lymphoma cannot be excluded). Multiple small nodules in both lungs, splenomegaly, possible left renal hypoplasia.

Based on the child's auxiliary examination results and following a hospital-wide consultation, the nature of the esophageal mass remains undetermined (lymphoma highly probable). Referral to General Surgery is recommended for further diagnostic procedures including bone marrow aspiration, lymph node biopsy, and endoscopic tissue biopsy. The child has congenital hypoplasia of the left kidney with normal renal function; observation is advised with ultrasound follow-up every 3–6 months. After discussion with the family, referral to Pediatric General Surgery for further management was decided.

**Figure 1:**
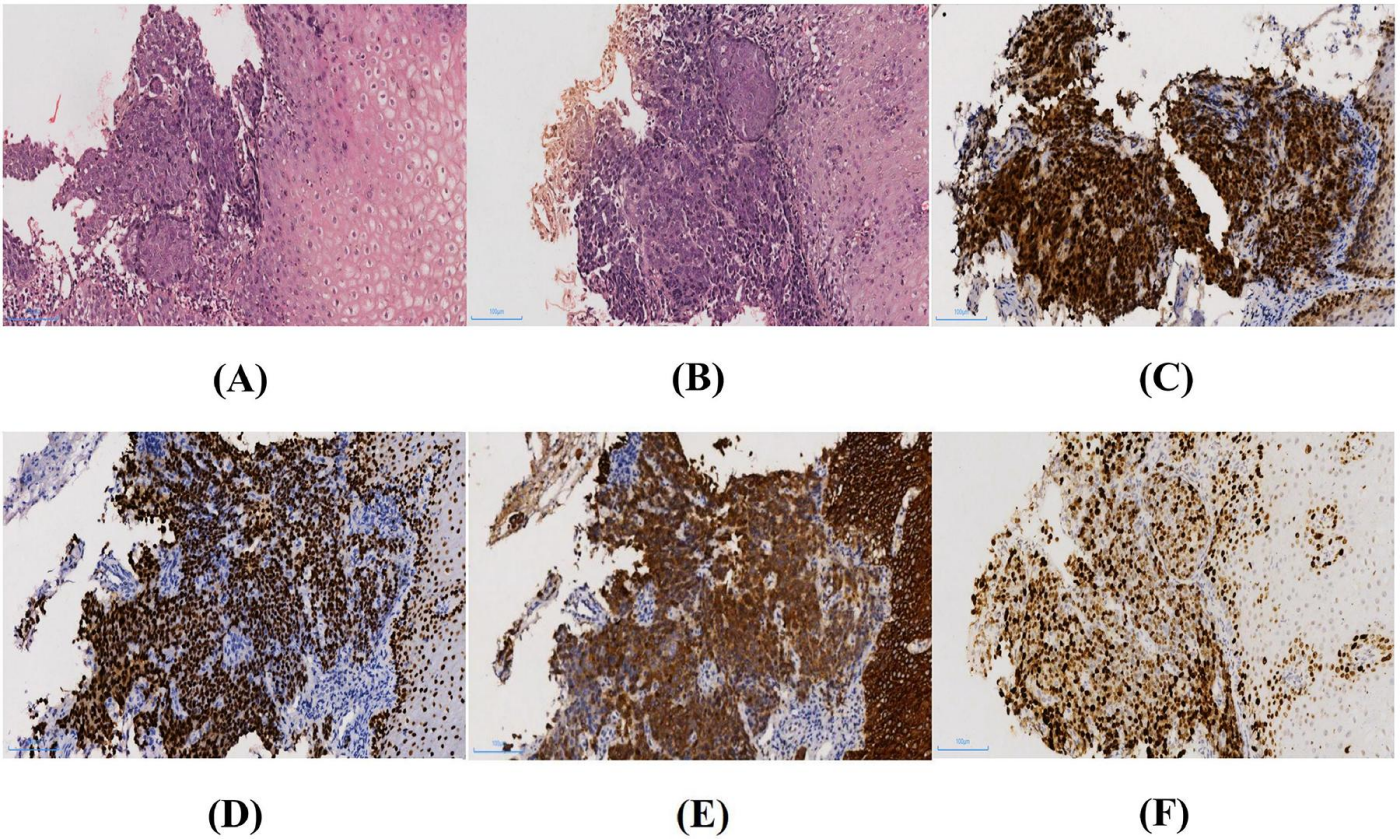
Pathological and immunohistochemical features of esophageal tumor in the patient.images were acquired at 200× magnification. The scale bar represents 100 µm. Original images were color balanced and cropped for presentation. No other digital alterations were performed. **(A,B)** Medium amplification shows infiltrative growth of atypical squamous cells with marked atypia by H&E. **(C,D)** Diffuse and strong in nuclear expression of P53 (up) and P63 (down). **(E)** CK5/6 showed diffuse membranous positivity. **(F)** The Ki-67 proliferation index was approximately 50%.

After being transferred to the general surgery department, the patient underwent a bone marrow aspiration cytology examination on March 2, 2020. The results showed no significant abnormalities and no evidence of abnormal bone marrow proliferation, ruling out the possibility of lymphoma. After ruling out surgical contraindications, the patient underwent “gastroscopy with tissue biopsy” on March 6, 2020. Under electronic fiberoptic endoscopy, extensive mucosal erosion and large ulcers were observed in the lower esophagus, covered with foul-smelling exudate. The mucosa appeared fragile and bled readily upon contact. Postoperative pathology was reported on March 11. Histopathological examination showed infiltrative nests of markedly atypical squamous cells with severe cytologic atypia, loss of polarity, and frequent mitoses, consistent with a diagnosis of esophageal squamous cell carcinoma (Figures 1A, B). Immunohistochemical analysis showed strong and diffuse nuclear expression of P53 (mutant-type) and P63 (Figures 1C, D), CK5/6 (Figure 1E) and CKpan were diffusely positive in membrane and cytoplasm, respectively. BCL-2 expression maybe focal, whereas CD56 was partially positive.There was marked negativity for CK7、SYN and LCA. The Ki-67 (Figure 1F) proliferation index was approximately 50%. Following consultation with external specialists and our hospital's multidisciplinary team, combined with the findings from tumor markers, PET-CT, and histopathology results, a diagnosis of squamous cell carcinoma of the lower esophagus. According to the 8th edition of the TNM staging system for esophageal squamous cell carcinoma, the tumor was classified as T1 (submucosal invasion without involvement of the muscularis propria), N2 (multiple regional lymph node metastases involving the mediastinal, paraesophageal, and retroperitoneal lymph nodes), and M0 (no distant metastasis), corresponding to stage IIIA. Although the primary tumor is confined to the submucosa, the presence of extensive lymph node metastases indicates that this represents a locally advanced disease. Considering the presence of extensive lymph node metastasis (N2) and poor nutritional status, the patient is not a candidate for surgery. Therefore, neoadjuvant chemotherapy was recommended as initial treatment.

**Figure 2:**
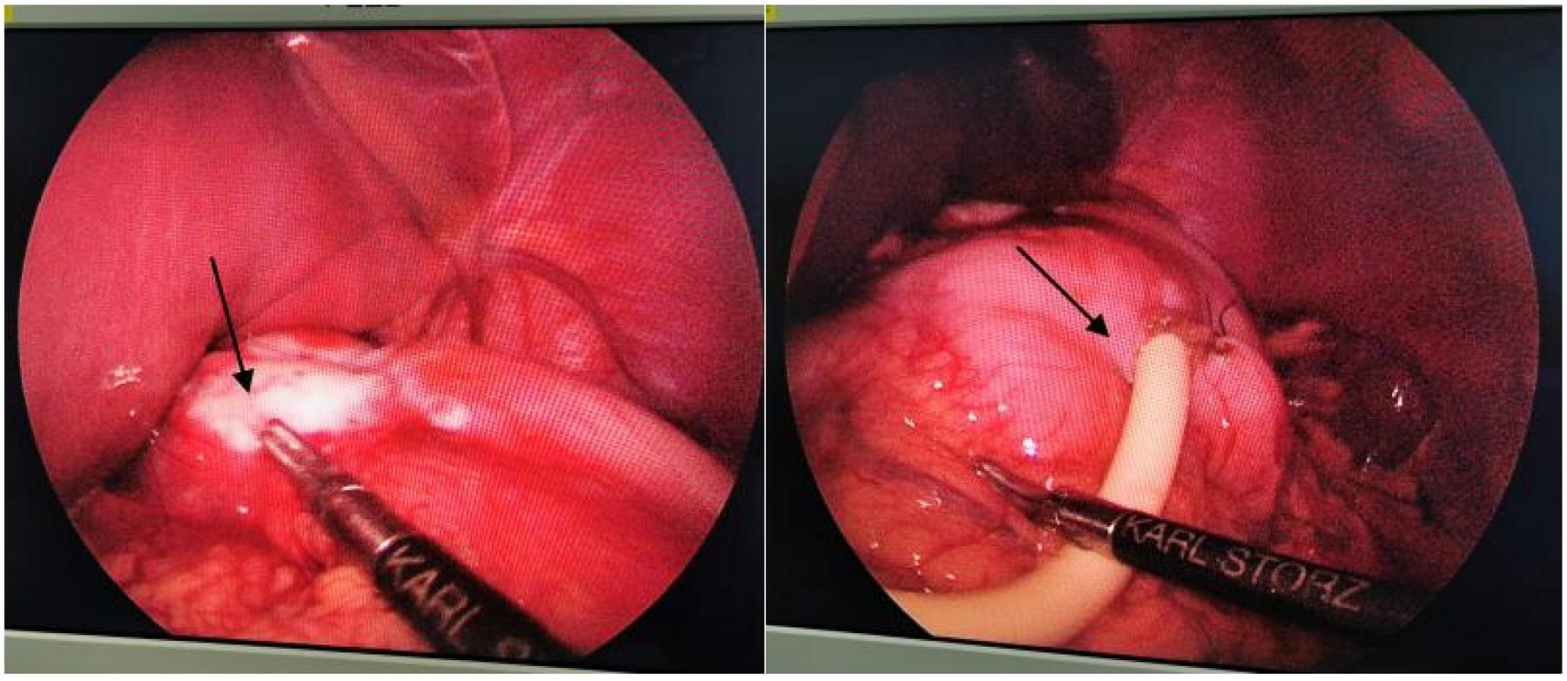
Tumor tissue at the junction of the esophagus and gastric fundus is visible during laparoscopy (left image, arrow). A gastric stoma was created at the greater curvature of the stomach during surgery (right image, arrow).

The patient's condition stabilized postoperatively. The family requested discharge against medical advice. After fully informing them of the diagnosis and relevant precautions, discharge procedures were completed. The patient was readmitted due to esophageal stricture, laparoscopic gastrostomy was performed on April 3, 2020 to relieve obstruction after ruling out surgical contraindications. Intraoperative findings revealed tumor tissue and the gastrostomy tube placed during the procedure (Figure 2). Postoperatively, the patient was placed on fasting with intravenous fluid support. Enhanced nutrition was provided via a specially formulated total parenteral nutrition (TPN) regimen to ensure adequate caloric intake. TPN formula: Medium/Long-chain Triglyceride Emulsion 100 mL + 10% Glucose Injection 400 mL + 50% Glucose Injection 140 mL + Soluble Vitamin Injection 1 vial + Liposoluble Vitamin Injection (I) 1 vial + 59 mL concentrated sodium chloride injection + 29 mL potassium chloride injection + 350 mL pediatric compound amino acid injection (18AA-1) + 10 mL multivitamin injection (I) + 20 mL glycerol phosphate sodium injection + 2.9 mL magnesium sulfate injection.

**Figure 3:**
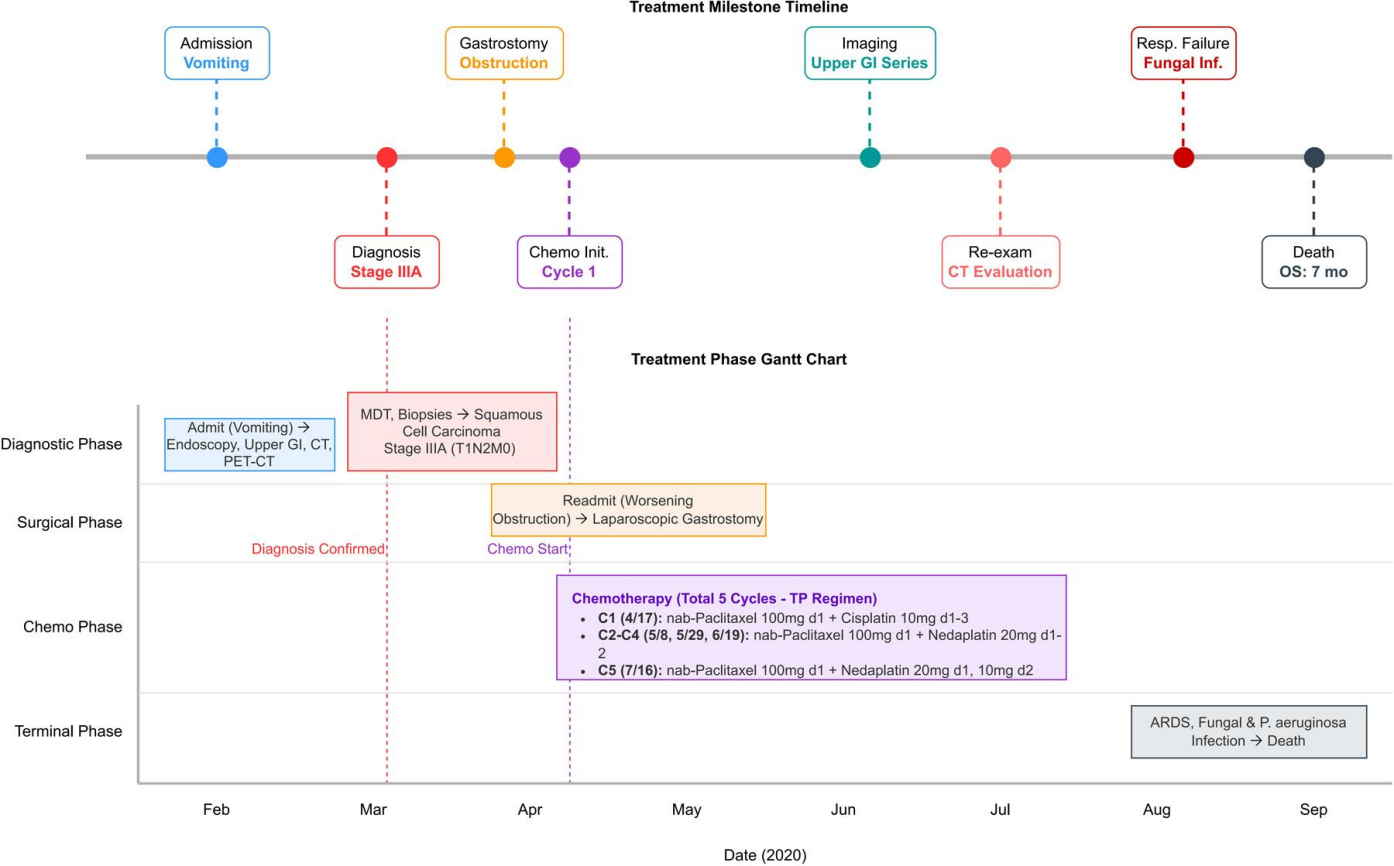
Comprehensive visualization of treatment course for pediatric lower esophageal squamous cell carcinoma.

After the pediatric patient's condition stabilized, chemotherapy was selected as the initial systemic therapy to control tumor progression, based on thorough discussion and comprehensive evaluation by our multidisciplinary team. The choice of the nab-paclitaxel plus platinum regimen was supported by its proven efficacy in adult esophageal squamous cell carcinoma and its alignment with CSCO guideline recommendations. On April 17, 2020, after ruling out contraindications to chemotherapy, the first chemotherapy session was administered with the following regimen: nab-paclitaxel 100 mg d1 + cisplatin 10 mg d1–3. Grade III gastrointestinal reactions occurred during chemotherapy, which improved after symptomatic treatment. On May 8, the chemotherapy regimen was adjusted to: nab-paclitaxel 100 mg d1 + nedaplatin 20 mg on days 1–2, supplemented with acid suppression, gastric protection, antiemesis, and other supportive therapies. On May 28, follow-up enhanced chest and abdominal CT showed stable disease (SD). After ruling out contraindications, two additional cycles of “nab-paclitaxel 100 mg on day 1 + nedaplatin 20 mg on days 1–2” were administered on May 29 and June 19. During this period, Grade I leukopenia (1.8 × 10^9^/L) developed, which improved after symptomatic management. Subsequently, long-term oral therapy with “Diyu Shengbai Tablets (0.2 g, three times daily)” was initiated to increase white blood cell count. Upper GI series on June 19 revealed esophageal-cardia segment stenosis presenting as incomplete obstruction, with the narrowed segment measuring approximately 3.3 cm in length. On July 7, contrast-enhanced chest and abdominal CT showed improvement in gastric wall thickening at the lower esophagus and cardia compared to previous imaging. Multiple enlarged lymph nodes were noted in the mediastinum, paraesophageal region, omental bursa, and retroperitoneum (enlarged and partially confluent compared to previous scans). Slightly hypointense nodules in the left lateral segment and right lobe of the liver were observed (reduced in size compared to previous scans). However, the patient continued to experience dysphagia, inability to eat, and frequent nausea and vomiting with no significant improvement in symptoms. Daily nutrition was provided via gastrostomy feeding and intravenous nutritional support. After excluding contraindications for chemotherapy, the TP regimen was resumed on July 16 with adjusted doses: nab-paclitaxel 100 mg on day 1 + nedaplatin 20 mg on day 1 and 10 mg on day 2. Concurrent supportive therapy included acid suppression for gastric protection and antiemetics. On August 13, the patient developed chest wall pain, predominantly on the left side (NRS score 2–3, paroxysmal exacerbations), with marked chest tightness and dyspnea after activity, relieved by oxygen therapy. Chest CT plain scan revealed multiple patchy opacities in both lungs, raising suspicion of fungal infection (metastatic tumors not excluded). Arterial blood gas analysis indicated acute respiratory distress syndrome and type I respiratory failure. Symptomatic antifungal therapy was administered for 2 weeks. On August 28, follow-up chest CT scan showed partial absorption and reduction of the original infection focus compared to previous imaging, while consolidation in the left lower lobe had significantly increased. Sputum culture identified Pseudomonas aeruginosa. Based on antimicrobial susceptibility testing, combined therapy with “Ceftazidime + Caspofungin” was initiated. Continuous pain management via an analgesic pump was concurrently administered. Subsequently, the patient's condition progressively deteriorated. The family decided to discharge the patient, and the child unfortunately passed away on September 9, 2020 (Figure 3).

## 3 Discussion

Malignant solid tumors of the gastrointestinal tract are extremely rare in children. At initial diagnosis, they are often already at an advanced stage, carry a poor prognosis, and present with nonspecific clinical manifestations. The early symptoms in this patient showed no significant difference from common gastrointestinal disorders such as functional disturbances. Conventional ultrasound and laboratory tests also yield no specific findings, complicating early diagnosis. Gastroscopy is suitable for children of all ages, enabling direct visualization of upper gastrointestinal lesions and mucosal biopsy sampling. This procedure provides diagnostic evidence for rare diseases and holds significant value in diagnosing pediatric upper gastrointestinal disorders. However, due to the extremely low incidence of esophageal cancer in children, mucosal biopsy is not routinely performed. In clinical practice, when conventional treatments for pediatric gastrointestinal disorders prove ineffective, endoscopy should be promptly employed to determine the cause and avoid diagnostic delays. Furthermore, biomarkers hold potential value in the early diagnosis of esophageal cancer and monitoring the progression of esophageal corrosive lesions. Studies have identified abnormal miRNA expression in pediatric esophageal cancer patients, with miR-374 and miR-574 implicated in esophageal tumorigenesis. Clarifying the association between miRNA expression and esophageal cancer may aid in elucidating the malignant transformation mechanism from corrosive stricture to neoplastic lesions (25). However, whether these biomarkers can serve as potential markers for early detection of pediatric esophageal cancer requires further validation. Currently, the diagnosis of esophageal cancer in children remains a significant challenge.

The incidence of pediatric solid tumors exhibits two distinct peaks: before age 3, embryonic tumors predominate, such as neuroblastoma and Wilms tumor; while before adolescence, hepatocellular carcinomas and thyroid cancers are more common (5–7). Among childhood malignancies, neuroblastoma, Wilms tumor, and hepatoblastoma are relatively frequent, with primary sites most commonly occurring in the intracranial, thoracic, bone, and soft tissue regions (8). Tumors within the digestive tract are extremely rare. Squamous cell carcinoma is more common in adults, with very few reports of esophageal cancer in children. In 1981, a case was reported of a 10-year-old female child with esophageal squamous cell carcinoma who died due to extensive liver metastasis (9); In 1995, a pediatric esophageal carcinoma case with concomitant esophagogastric fistula was reported. Pathological diagnosis revealed esophageal papillary adenocarcinoma with a tent-shaped structure, involving the entire wall thickness. Metastatic lesions were observed in the esophageal adventitia and paragastric lymph nodes. The patient died from respiratory failure following surgical intervention (10). In 2012, a 10-year-old female patient with poorly differentiated squamous cell carcinoma was reported, though specific treatment details and prognosis remain unclear (11). Another case described a 7-year-old male with esophageal leiomyosarcoma who underwent Phemister procedure (thoracic esophagectomy with esophagogastrostomy). No adjuvant chemoradiotherapy was administered postoperatively, and the patient remained recurrence-free at 3-year follow-up. The investigators suggested that esophagectomy should be performed whenever possible in pediatric leiomyosarcoma to maximize therapeutic benefit. Radiotherapy and chemotherapy may be effective for unresectable, recurrent, or metastatic lesions (12). In 2015, a case of familial esophageal cancer in a pediatric patient was reported. The family history included hamartomas and germline PTEN frameshift and SMAD7 missense mutations. The patient, a Caucasian male, was diagnosed with juvenile polyposis syndrome (JPS). At age 41, he developed distal esophageal adenocarcinoma and multiple juvenile polyps in the stomach, duodenum, and colon with lymph node metastasis. After esophagectomy and adjuvant therapy, he was diagnosed again at age 54 with squamous cell carcinoma at the esophagogastric anastomosis. He died at age 57 following palliative chemoradiotherapy. His son was diagnosed with distal esophageal adenocarcinoma with lymph node metastasis at age 33. He underwent esophagectomy combined with neoadjuvant chemoradiotherapy; prognosis is unknown (13).

Reports on esophageal cancer in children remain extremely scarce, with only two documented cases of esophageal squamous cell carcinoma. Combined with the case presented herein, all reported cases exhibit poor prognosis. While advancements in medical technology have significantly improved the diagnosis and treatment of various acute and chronic diseases, research on pediatric tumors—particularly malignant tumors—lags behind. Malignant solid tumors in children have become one of the leading causes of childhood mortality. Pediatric esophageal squamous cell carcinoma remains exceptionally rare, with no established diagnostic or treatment protocols currently available. However, China is a high-incidence country for esophageal cancer, accounting for approximately half of all new cases annually. Male incidence and mortality rates are particularly elevated, ranking fifth and fourth globally, respectively (14). Adult esophageal cancer often presents with atypical symptoms, making early detection challenging. Most patients present at intermediate or advanced stages, where direct surgery is difficult and effective drug treatments are limited. Radiotherapy demonstrates significant efficacy, and multidisciplinary comprehensive treatment is key to improving prognosis. The 2020 CSCO Esophageal Cancer Diagnosis and Treatment Guidelines clearly define endoscopic resection and surgical indications for early-stage esophageal squamous cell carcinoma, emphasize the importance of preoperative induction therapy and postoperative adjuvant therapy, and introduce PD-1 monoclonal antibody therapy as a new second-line treatment for distant metastatic esophageal squamous cell carcinoma. Extensive research confirms that postoperative adjuvant radiotherapy improves survival rates in patients with lymph node metastasis, with concurrent chemoradiotherapy offering greater survival benefits than adjuvant radiotherapy alone (15, 16). For resectable esophageal cancer, the synergistic combination of neoadjuvant chemotherapy and surgery yields significant clinical efficacy. The recommended timing for surgery after neoadjuvant therapy is 4–8 weeks after completion of chemoradiotherapy or 3–6 weeks after completion of chemotherapy, provided the patient's physical condition permits (17, 18). It should be noted that adjuvant treatment outcomes differ between squamous cell carcinoma and adenocarcinoma of the esophagus. Adjuvant chemotherapy prolongs disease-free survival in squamous cell carcinoma patients but does not significantly improve overall survival. Given that squamous cell carcinoma predominates among Chinese esophageal cancer patients, these findings hold significant clinical implications (19, 20).

Based on the case presented herein and analysis of relevant literature, pediatric esophageal cancer treatment may draw upon adult multidisciplinary comprehensive treatment models. However, it is essential to fully account for the physiological differences between children and adults. Key diagnostic and therapeutic considerations are outlined below:

### 3.1 Treatment selection

Literature reports indicate that squamous cell carcinoma predominates in pediatric esophageal cancer (accounting for 60%), with 48% of patients presenting metastatic disease at diagnosis and a poor prognosis. Surgery or chemotherapy alone yields limited efficacy (3). For resectable tumors, a comprehensive approach combining neoadjuvant chemoradiotherapy with surgical resection has been shown to improve survival in adult esophageal cancer and has been attempted in a few pediatric case reports. For instance, a 13.5-year-old patient with esophageal squamous cell carcinoma underwent preoperative chemoradiotherapy followed by tumor resection, with no tumor recurrence observed during a two-year follow-up (21). A 14-year-old patient with esophageal adenocarcinoma achieved disease-free survival for 5 years during follow-up after receiving neoadjuvant chemotherapy, surgery, and postoperative chemoradiotherapy (3). However, direct evidence supporting this strategy in children remains extremely limited, as no large-scale retrospective studies are available. The present case, in which the patient was not eligible for curative surgery or radiotherapy, does not provide direct support for this approach. For unresectable or advanced tumors, palliative chemoradiotherapy can alleviate symptoms such as dysphagia and pain while controlling tumor progression. Some pediatric patients experience improvement in bleeding and obstruction symptoms following palliative radiotherapy. Additionally, cisplatin combined with 5-fluorouracil is a commonly used chemotherapy regimen for esophageal cancer. Taxanes and irinotecan may also be considered as combination options. Novel targeted therapies (e.g., trastuzumab, pembrolizumab) have demonstrated favorable efficacy in adults (22, 23), and their potential application in pediatric cases warrants future exploration. Given the lack of pediatric-specific evidence, treatment decisions for pediatric esophageal squamous cell carcinoma must be individualized within a multidisciplinary framework. In carefully selected cases where tumor downstaging is considered achievable, neoadjuvant chemoradiotherapy followed by surgery—an approach derived from adult guidelines—may be cautiously explored. However, it must be emphasized that their feasibility, safety, and efficacy require further investigation.

### 3.2 Key points of surgical treatment

There is currently no definitive diagnostic and treatment protocol for pediatric esophageal cancer. Clinically, adult treatment principles are often referenced: Endoscopic mucosal resection or submucosal resection may be performed for early-stage tumors. For resectable tumors with muscle or deeper layer invasion, esophagectomy combined with lymph node dissection is the primary treatment approach. For children with stage T2 tumors without lymph node metastasis, primary esophagectomy combined with adjuvant chemotherapy may be considered. Children with T2 tumors with lymph node metastasis, T3, or T4a tumors should undergo neoadjuvant chemoradiotherapy first, followed by surgical resection after tumor downstaging. T4b tumors (invading the heart, major vessels, trachea, etc.) are unresectable (4, 22). Due to the unique anatomical location of cervical esophageal cancer, surgical feasibility requires careful assessment given the high technical difficulty (3). In pediatric patients, any decision regarding curative surgery or neoadjuvant therapy should be made cautiously after multidisciplinary discussion, taking into account children's unique physiological reserve, long-term quality of life, and the late effects of chemoradiotherapy. In the present case, histopathological examination confirmed that the tumor was confined to the submucosal layer (T1). Nevertheless, multiple regional lymph node metastases were identified (N2), resulting in a stage IIIA classification according to the 8th edition TNM staging system. The patient had no indications for curative surgery; therefore, only palliative gastrostomy and systemic chemotherapy were administered. The lack of opportunity for curative surgery was a major factor contributing to the poor prognosis.

### 3.3 Importance of supportive care

Pediatric esophageal cancer patients often present with dysphagia, weight loss, anemia, and other symptoms. Supportive care improves quality of life and lays the foundation for antitumor therapy. Severe dysphagia can be alleviated through balloon dilation or stent placement to relieve obstruction. For patients unable to eat orally, percutaneous gastrostomy provides nutritional support. Concurrently, complications such as anemia and infection require active correction (24). In this case, endoscopic stent placement was technically challenging due to the tumor's location in the lower esophagus and its morphological characteristics. Given the patient's poor nutritional status, performing a gastrostomy provided a reliable enteral nutrition route without imposing an immediate risk of radiotherapy, thereby improving nutritional intake and enabling multiple cycles of chemotherapy. However, treatment ultimately failed due to severe infection and tumor progression.

While adult esophageal cancer research is well-established with continuously updated treatment protocols, pediatric esophageal cancer lacks standardized guidelines due to its rarity. Further accumulation of case data is needed to develop treatment models tailored to children. Overall survival rates in children can exceed 80%, significantly higher than in adults (e.g., the cure rate for childhood acute lymphoblastic leukemia reaches 80%, compared to only 30%–40% in adults). The diagnosis and treatment of pediatric malignant solid tumors necessitate close collaboration among multiple disciplines, including surgery, internal medicine, oncology, radiology, and pathology. Adopting a multidisciplinary integrated approach is crucial for improving treatment efficacy and prognosis. In the future, for children with confirmed or suspected esophageal cancer, a multidisciplinary team may consider evaluating the potential role of neoadjuvant chemoradiotherapy followed by surgery, aiming to prolong survival and enhance treatment outcomes, drawing on adult protocols with careful individualization.

## Data Availability

The original contributions presented in the study are included in the article/Supplementary Material, further inquiries can be directed to the corresponding author.

## References

[1] Hou X Li S Wu C Li K Li Q Li X Clinical analysis of gastrointestinal malignant solid tumors in children and adolescents. Chin J Pract Pediatr. (2018) 33(12):928–32. 10.3760/cma.j.issn.2095-428X.2018.12.012

[2] Chen K Jiang H Xi Z Chen R Zhang R Xie H Analysis of the incidence of solid tumors in children. In: Zhejiang Medical Association, editor. Proceedings of the 17th National Congress of Pediatrics of the Chinese Medical Association. Jiaxing, Zhejiang: Pediatrics Branch of Chinese Medical Association: Chinese Medical Association (2012). volume 1: p. 2.

[3] Coşkun Ç Kurucu N Kutluk T Oguz B Orhan D Cengiz M Esophageal carcinoma in children: report of 2 cases and a review of the literature. J Pediatr Hematol Oncol. (2024) 46(1):e94–9. 10.1097/MPH.0000000000002772 37878545

[4] Jamel S Tukanova K Markar S. Detection and management of oligometastatic disease in esophageal cancer and identification of prognostic factors: a systematic review. World J Gastrointest Oncol. (2019) 11(9):741–9. 10.4251/wjgo.v11.i9.741 31558978 PMC6755111

[5] Davesa SS Blot WT Stone BJ Miller BA Tarone RE Fraumeni JF. Recent cancer trends in the United States. J Natl Cancer Inst. (1995) 87:175–9. 10.1093/jnci/87.3.175 7707404

[6] Gu S. Experience in diagnosis and treatment of retroperitoneal tumors in children. J Zunyi Med Univ. (2003) 26(3):268–9. 10.3969/j.issn.1000-2715.2003.03.029

[7] Gurney JG Severson RK Davis S Robison LL. Incidence of cancer in children in the United States. Sex, race, and 1-year age-specific rates by histologic type. Cancer. (1995) 75:2186–95. 10.1002/1097-0142(19950415)75:8<2186::aid-cncr2820750825>3.0.co;2-f 7697611

[8] Tang J Li Z. Diagnosis and Treatment of Pediatric Tumors. Beijing: People’s Military Medical Press (2011). p. 301–406.

[9] Sun M Ma X. A case report of esophageal cancer in a child. Tumor. (1981) 1(06):37.

[10] Wang H Han T Yang S Ding H Chen Z. A case of esophageal cancer with esophageal-left bronchus Fistula in a child. Chin J Thorac Cardiovasc Surg. (1995) 11(06):354. 10.3760/cma.j.issn.1001-4497.1995.06.126

[11] Wang L Yu Y Liu C Shi L. A case of esophageal cancer in a child. Chin J Misdiagn. (2012) 12(03):632.

[12] Wang W-x Desai G Wen L Ye MF Sun QR Liu WJ Pediatric esophageal leiomyosarcoma: a case report. J Pediatr Surg. (2011) 46:1646–50. 10.1016/j.jpedsurg.2011.04.003 21843737

[13] Sherman SK Maxwell JE Qian Q Bellizzi AM Braun TA Iannettoni MD Esophageal carcinoma in a familial hamartomatous syndrome with germline PTEN frameshift and SMAD7 missense mutations. Cancer Genet. (2015) 208:41–6. 10.1016/j.cancergen.2014.11.002 25554686 PMC4355394

[14] Editorial Department. Recommended Reading from tumor prevention and treatment: interpretation of the 2020 Chinese society of clinical oncology guidelines for esophageal cancer diagnosis and treatment. Tumor Prevention and Treatment. (2021) 34(02):107.

[15] Zheng Y Wang Z Yan C Yan M Hou Z Zheng R Randomized controlled trial protocol for perioperative S-1 combined with oxaliplatin plus apatinib and camrelizumab in patients with resectable locally advanced gastric cancer or gastresophageal junction adenocarcinoma. Ann Transl Med. (2020) 8(24):1684. 10.21037/atm-20-7802 33490196 PMC7812167

[16] Yu S Zhang W Ni W Xiao Z Wang Q Zhou Z Propensity score matching analysis comparing long-term survival outcomes between surgery alone and postoperative therapy in patients with lymph node-positive or stage III esophageal squamous cell carcinoma after R0 esophagectomy. Radiother Oncol. (2019) 140:159–66. 10.1016/j.radonc.2019.06.020 31302346

[17] Wang C Han Y. Interpretation of the 2020 Chinese society of clinical oncology guidelines for diagnosis and treatment of esophageal cancer. Cancer Prevention and Treatment. (2020) 33(04):285–90. 10.3969/j.issn.1674-0904.2020.04.001

[18] Yao W. Clinical efficacy of neoadjuvant chemotherapy combined with surgery in the treatment of esophageal cancer. J Aerosp Med. (2021) 32(05):567–8. 10.3969/j.issn.2095-1434.2021.05.031

[19] Cunningham D Allum WH Stenning SP Thompson JN Van de Velde CJH Nicolson M Perioperative chemotherapy versus surgery alone for respectable gastroesophageal cancer. N Engl J Med. (2006) 355(1):11–20. 10.1056/NEJMoa055531 16822992

[20] Zhang L Li W Lyu X Song Y Mao Y Wang S Adjuvant chemotherapy with paclitaxel and cisplatin in lymph node-positive thoracic esophageal squamous cell carcinoma. Chin J Cancer Res. (2017) 29(2):149–55. 10.21147/j.issn.1000-9604.2017.02.08 28536494 PMC5422417

[21] Alami A Outtaleb FZ Houjami M Chekrine T Bouchbika Z Benchakroun N A rare cause of dysphagia in children: squamous cell carcinoma of the esophagus: a case report and review of literature. Gastroenterology and Hepatology Open Access. (2021) 12(2):28–9. 10.15406/ghoa.2021.12.00452

[22] Kurtom SS Kaplan BJ. Esophagus and gastrointestinal junction tumors. Surg Clin North Am. (2020) 100(3):507–21. 10.1016/j.suc.2020.02.003 32402297

[23] Ku GY. Systemic therapy for esophageal cancer: chemotherapy. Chin Clin Oncol. (2017) 6(5):49. 10.21037/cco.2017.07.06 29129089

[24] Layke JC Lopez PP. Esophageal cancer: a review and update. Am Fam Physician. (2006) 73(12):2187–94. 16836035

[25] de Oliveira Jr WE Felix TF do Vale PG Lapa RML Severino FE Terra SA MicroRNA expression profiles in the esophagus of children with caustic stenosis: a pathway towards esophageal cancer?. J Pediatr Surg. (2020) 55:2144–9. 10.1016/j.jpedsurg.2020.02.009 32111433

